# Supplementary material for: Identification of photoperiod-induced specific miRNAs in the adrenal glands of Sunite sheep (Ovis aries)
Source: Front Vet Sci. 2022 Jul 22;9:888207. doi: 10.3389/fvets.2022.888207 (PMC9354845; doi:10.3389/fvets.2022.888207)
Supplement: Supplementary file 1 [file Data_Sheet_1.ZIP › Supplementary file/miRDeep2 file.zip!/miRDeep2 file/Novel_Structure/LP42A3/Novel_448.pdf]

Provisional ID	:	NC_019460.2_6766
Score total	:	0.6
Score for star read(s)	:	-1.3
Score for read counts	:	0
Score for mfe	:	0.3
Score for randfold	:	1.6
Score for cons. seed	:	
Total read count	:	38
Mature read count	:	38
Loop read count	:	0
Star read count	:	0

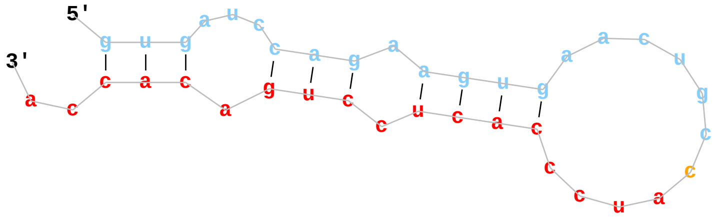

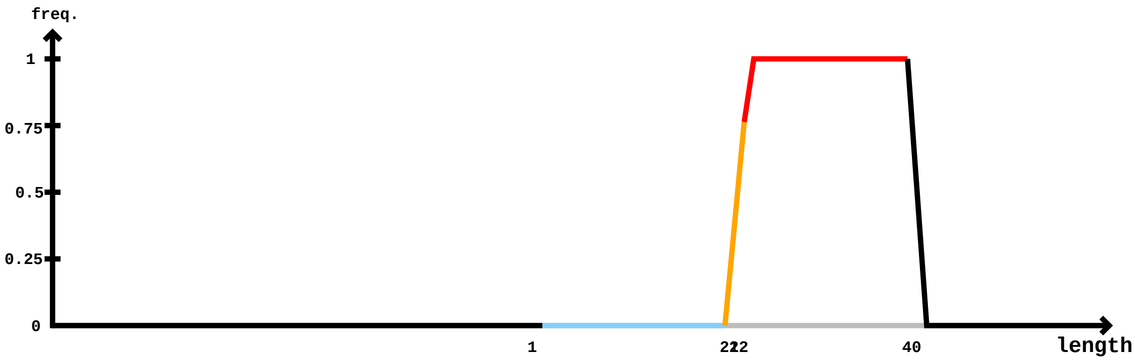

Star Mature

```
5'- accauccuuguaacgcugagauccuaaagagcugcauccucagcuacaggugauccagaagugaacugccaucccacuccugacaccaagcaaagccugccaagggug   -3'    exp
    ..((((((((....((((((.................))))))....((((...(((.((((...........)))).))).))))..(((.....)))))))))))    reads      mm         sample
    ......................................................................aucccacuccugacacca....................    29         0          seq
    .......................................................................ucccacuccugacacca....................    9          0          seq
```
